# Is attention branch network effective in classifying dental implants from panoramic radiograph images by deep learning?

**DOI:** 10.1371/journal.pone.0269016

**Published:** 2022-07-27

**Authors:** Shintaro Sukegawa, Kazumasa Yoshii, Takeshi Hara, Futa Tanaka, Katsusuke Yamashita, Tutaro Kagaya, Keisuke Nakano, Kiyofumi Takabatake, Hotaka Kawai, Hitoshi Nagatsuka, Yoshihiko Furuki

**Affiliations:** 1 Department of Oral and Maxillofacial Surgery, Kagawa Prefectural Central Hospital, Takamatsu, Kagawa, Japan; 2 Department of Oral Pathology and Medicine, Graduate School of Medicine, Dentistry and Pharmaceutical Sciences, Okayama University, Okayama, Japan; 3 Department of Intelligence Science and Engineering, Graduate School of Natural Science and Technology, Gifu University, Gifu, Japan; 4 Department of Electrical, Electronic and Computer Engineering, Faculty of Engineering, Gifu University, Gifu, Gifu, Japan; 5 Center for Healthcare Information Technology, Tokai National Higher Education and Research System, Gifu, Gifu, Japan; 6 Polytechnic Center Kagawa, Takamatsu, Kagawa, Japan; Thamar University, Faculty of Dentistry, YEMEN

## Abstract

Attention mechanism, which is a means of determining which part of the forced data is emphasized, has attracted attention in various fields of deep learning in recent years. The purpose of this study was to evaluate the performance of the attention branch network (ABN) for implant classification using convolutional neural networks (CNNs). The data consisted of 10191 dental implant images from 13 implant brands that cropped the site, including dental implants as pretreatment, from digital panoramic radiographs of patients who underwent surgery at Kagawa Prefectural Central Hospital between 2005 and 2021. ResNet 18, 50, and 152 were evaluated as CNN models that were compared with and without the ABN. We used accuracy, precision, recall, specificity, F1 score, and area under the receiver operating characteristics curve as performance metrics. We also performed statistical and effect size evaluations of the 30-time performance metrics of the simple CNNs and the ABN model. ResNet18 with ABN significantly improved the dental implant classification performance for all the performance metrics. Effect sizes were equivalent to “Huge” for all performance metrics. In contrast, the classification performance of ResNet50 and 152 deteriorated by adding the attention mechanism. ResNet18 showed considerably high compatibility with the ABN model in dental implant classification (AUC = 0.9993) despite the small number of parameters. The limitation of this study is that only ResNet was verified as a CNN; further studies are required for other CNN models.

## 1. Introduction

Dental implants are a long-term predictable treatment option for defect prostheses [1]. In particular, the osseointegration type root form implants currently have high predictability and are commonly used [2, 3]. With the increase in reliability and success rate of dental implant treatment, the number of patients treated with dental implant prostheses is increasing.

On the other hand, there have been reports of complications as the number of dental implants increases [4, 5]. Complications related to dental implants are classified into biological and mechanical complications [6, 7]; therefore, it is important to understand the characteristics of each type of implant such as the shape of implant fixture, surface texture, type of abutment joint, and fixing screw. It is necessary to remove the implant when it becomes difficult to continue using it owing to complications. Although various devices are available as removal tools [8], understanding the type of implant is necessary for easy removal. Unfortunately, it has also been reported that implant removal is required if continuous maintenance is not possible due to difficulty in identifying the implant brand type [9]. Therefore, it is important to accurately identify the types of dental implants.

In recent years, studies using a classifier in deep learning to identify the type of implant have been reported [10–14]. Many of these studies employ convolutional neural networks (CNNs), which are more accurate owing to a number of parameters that keep deep layers. However, increasing the number of parameters in the deep layers leads to the burden of calculation cost. By adding another structure to the CNN structure [15] or changing the structure of the CNNs [16], various developments have been made, such as achieving the same accuracy with a small number of parameters.

The attention mechanism, which is a means of determining which part of the forced data is emphasized, has attracted attention in various fields of deep learning in recent years. The attention mechanism had been incorporated into a recurrent-neural-network-based model used in natural language processing called Seq2Seq and has received a lot of attention [17]. After that, it was used in advanced models such as Transformer [18] proposed for machine translation and BERT [19] based on it, and achieved high accuracy in various natural language processing tasks. Recently, the image attention mechanism has also been used in image recognition techniques. Attention branch network (ABN) [20] is attracting attention because it can recognize with high accuracy which area of the feature map output by CNNs should be focused on by the attention mechanism. Therefore, we predicted that higher accuracy could be achieved by adding ABN to the implant brand identification classification using deep learning. The purpose of this study was to evaluate the accuracy of ABN for implant classification accuracy using CNNs.

## 2. Materials and methods

### 2.1. Study design

The purpose of this study is to evaluate the effect of adding ABN to a deep learning model using CNNs on implant classification performance. This is a study based on deep learning using CNNs and supervised learning was adopted.

### 2.2. Ethics statement

This study was approved by the Institutional Review Board of Kagawa Prefectural Central Hospital (approval number; 1080). The Institutional Review Board, which reviewed our study, waived the need for personal informed consent because it featured a non-interventional retrospective design and all data was anonymized and analyzed.

### 2.3. Data set and pre-processing

There were two types of panoramic X-ray equipment, AZ3000CMR and Hyper-G CMF (ASAHIROENTGEN IND. Co. Ltd., Kyoto, Japan). Digital dental panoramic radiographs of patients were used. All digital image data were converted to a tagged image file format (TIFF) (2964 × 1464, 2804 × 1450,2694×1450, or 2776 × 1450 pixels) from the Kagawa Prefectural Central Hospital Picture Archiving and Communication Systems (PACS) (HOPE Dr ABLE-GX, FUJITSU Co., Tokyo, Japan). The dental implant brand was labeled based on the electronic medical record and our dental implant use ledger. A dataset of 10191 manually cropped image segments, each focused on dental implants, was generated from the original collection of digital panoramic radiographs taken.

Dental implant data included all data from various stages of treatment, such as implant fixtures, fixtures with healing abutments, interim prostheses, and final prostheses. In preparation for deep learning analysis, the panoramic X-ray image data output from PACS were imported into Photoshop Elements (Adobe Systems, Inc., San Jose, CA, USA) and were cropped to fit all dental implant instruments. The cropped images were saved in portable network graphics (PNG) format. The oral and maxillofacial surgeons who performed the image cropping as pre-processing were unaware of the brand of implant used for each patient.

### 2.4. Classification of dental implant brand

The 13 most used dental implant brands at Kagawa Prefectural Central Hospital were selected. The 13 types of dental implant systems that were the subject of this study are shown in Table 1. Table 1 shows the types of dental implant brands and the number of corresponding datasets. Table 2 shows the distribution of treatment stages by implant brand.

**Table 1:** Summary of thirteen types of dental implant systems.

Abbreviated name	Implant name	Company	Diameter (mm)	Length (mm)
Full OSSEOTITE 4.0	Full OSSEOTITE Tapered Certain	Zimmer Biomet, Florida, USA	4.0	8.5, 10, 11, 11.5
Astra EV 4.2	Astra Tech Implant System OsseoSpeed EV	Dentsply IH AB, Molndal, Sweden	4.2	9, 11
Astra TX 4.0	Astra Tech Implant System OsseoSpeed TX	Dentsply IH AB, Molndal, Sweden	4.0	8, 9, 11
Astra TX 4.5	Astra Tech Implant System OsseoSpeed TX	Dentsply IH AB, Molndal, Sweden	4.5	9, 11
Astra MicroThread 4.0	Astra Tech Implant System MicroThread	Dentsply IH AB, Molndal, Sweden	4.0	8, 9, 11
Astra MicroThread 4.5	Astra Tech Implant System MicroThread	Dentsply IH AB, Molndal, Sweden	4.5	9, 11
Brånemark Mk III 4.0	Brånemark System Mk III TiUnite	Nobelbiocare, Göteborg, Sweden	4.0	8.5, 10, 11.5
FINESIA 4.2	FINESIA BL HA TP	Kyocera Co., Kyoto, Japan	4.2	8, 10
POI EX 42	POI EX System	Kyocera Co., Kyoto, Japan	4.2	8, 10
Replace Select Tapered 4.3	Replace Select Tapered	Nobel Biocare, Göteborg, Sweden	4.3	8, 10, 11.5
Nobel Replace CC 4.3	Nobel Replace Conical Connection	Nobel Biocare, Göteborg, Sweden	4.3	8, 10, 11.5
Straumann Tissue 4.1	Standard Plus Implant Tissue Level	Straumann Group, Basei, Switzerland	4.1	8, 10
Straumann Bone Level 4.1	Standard Plus Implant Bone Level	Straumann Group, Basei, Switzerland	4.1	8, 10

**Table 2:** Distribution of implant brands used in the study.

Implant bland	Treatment status			
	Fixture	Fixture+ab	Prosthesis	Total
Full OSSEOTITE 4.0	279	25	123	427
Astra EV 4.2	350	307	188	845
Astra TX 4.0	1412	504	604	2520
Astra TX 4.5	523	158	433	1114
Astra MicroThread 4.0	337	82	285	704
Astra MicroThread 4.5	220	94	66	380
Brånemark Mk III 4.0	275	52	28	355
FINESIA 4.2	137	146	56	339
POI EX 42	95	109	177	381
Replace Select Tapered 4.3	302	178	136	616
Nobel Replace CC 4.3	1089	233	277	1599
Straumann Tissue 4.1	225	288	142	655
Straumann Bone Level 4.1	94	119	43	256

### 2.5. CNNs model architecture

ResNet is a neural network model proposed by Microsoft Research in 2015 [21]. In image recognition, it has been known that increasing the number of layers of a CNN generally results in the acquisition of higher-dimensional features. However, there was a problem that performance deteriorated due to loss of gradient when simply adding many layers. The residual network solved the gradient loss problem by introducing a shortcut connection and enabled the construction of CNNs with deep layers. The representative ResNet models, ResNet18, 50, and 152, were used in this study. Table 3 shows the number of parameters for each simple ResNet model.

**Table 3:** Number of parameters for simple ResNet model and ResNet with ABN model.

	Trainable parameters	Non-trainable parameters	Total parameters
**ResNet18**	11,717,584	7,942	11,725,526
**ResNet18+ABN**	13,491,801	8,456	13,500,257
**ResNet50**	25,611,984	45,574	25,657,558
**ResNet50+ABN**	30,959,254	53,634	31,012,888
**ResNet152**	60,247,760	143,878	60,391,638
**ResNet152+ABN**	65,644,182	151,938	65,796,120

ABN; Attention Branch Network (ABN).

To construct an efficient model, fine-tuning was used to re-train some of the weights of the existing trained models.

Therefore, all ResNet models have adopted fine-tuning using the ImageNet database. The deep learning classification task process was implemented using Keras (version 2.2.4), TensorFlow (version 1.15.2), and the Python language (version 3.7.10).

### 2.6. ABN model architecture

The ABN used in this study consists of three modules including feature extractor, attention branch, and perception branch. Table 3 shows the number of parameters for each ResNet with ABN model. In the ABN model, feature extraction was performed using the part of ResNet excluding the fully connected layer. Subsequently, the extracted features were connected to the ABN and attention mechanism as feature maps.

The main roles of each module are as follows.

*Feature extractor*: Module consisting of multiple convolution layers for extracting feature maps.
*Attention branch*: Module that outputs the class activation mapping (CAM) based attention position
*Perception branch*: A module that outputs class probability values by using features and attention maps for convolution layers.

#### 2.6.1. Attention branch

In this study, CAM with K = 13 classes consists of K × 3 × 3 convolutional layer, global average pooling (GAP), and a full concatenation layer. K × 3 × 3 convolutional layer outputs a feature map showing the gaze area in a particular class. This feature map is down sampled to K × 1 × 1 by GAP. The attention map in each class was visualized by weighting it with the joint weights of the fully connected layer. Instead of a fully connected layer for continuous learning, a convolutional layer was introduced in CAM. In this study, a 3 × 3 convolutional layer was introduced instead of an all-combining layer because the network holding a fully connected layer of VGG-Net was used as a baseline.

The top layer is built on a CAM base consisting of convolutional layers and GAPs to generate attention maps. However, since CAM uses the weights of all the learned convolutional layers to generate the attention map, it is not possible to generate the attention map during the training process. To solve this problem, we constructed a K × 1 × 1 convolutional layer instead of a fully connected layer.

**Fig 1:**
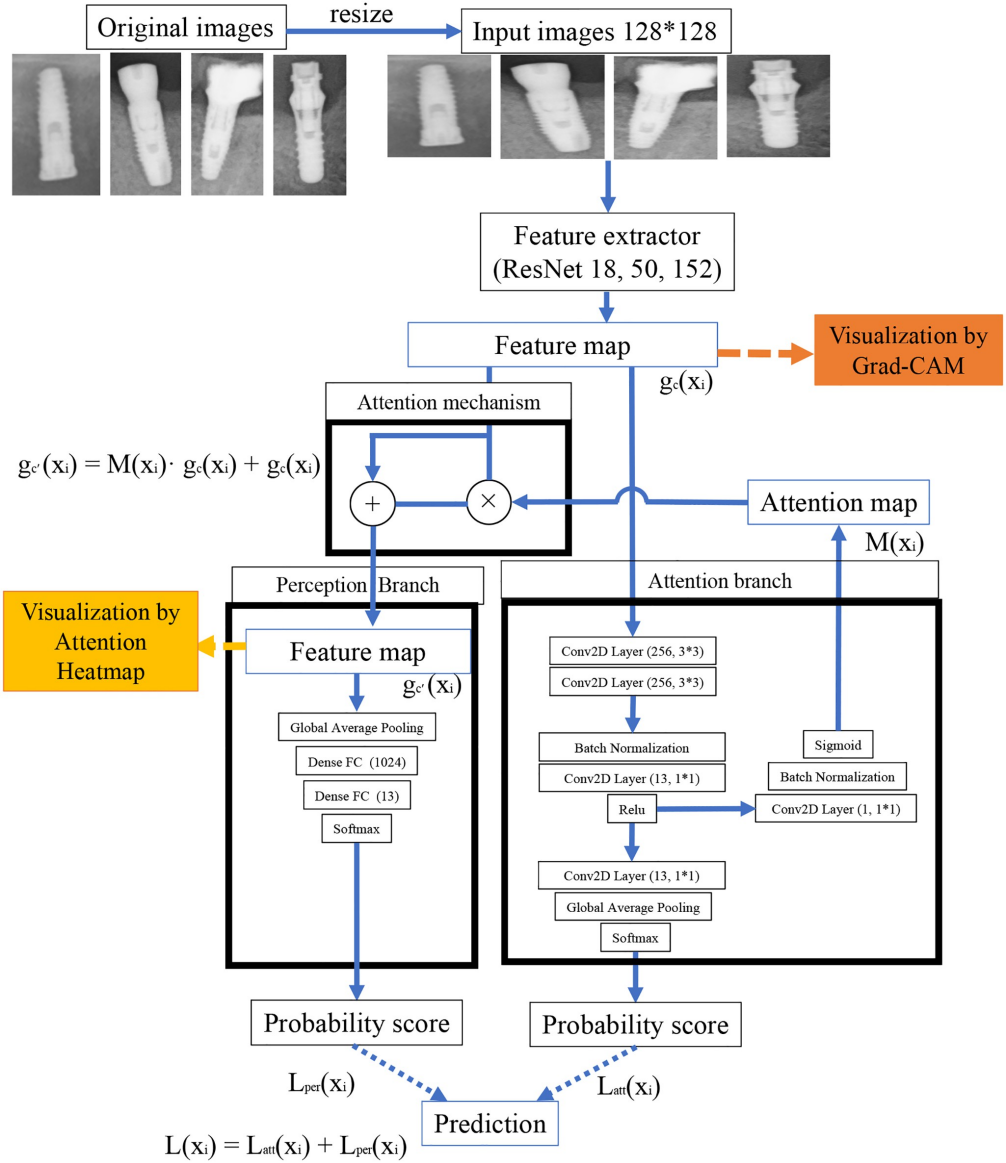
Schematic diagram of the attention branch network used in this study.

This convolutional layer mimics the final fully connected layer of CAM in the forward propagation process. After the convolutional layer, the attention branch generated the probability values of the classes by using the GAP return values together with the softmax function. Then, to aggregate the K feature maps, these feature maps were convolved by 1×1×1 convolutional layers to generate feature maps. We used the feature maps standardized by the sigmoid function as the attention maps for the attention mechanism. Fig 1 shows a schematic overview of the ABN model.

#### 2.6.2. Perception branch

The perception branch outputs the final probability value of each implant brand using the feature map by attention and feature extractor. The attention map was applied to the feature map by the attention mechanism. We introduced a calculation formula that emphasizes the feature map of the region where the attention map strongly responded and prevent the feature map from disappearing even in the zero region of the attention map.


$${\text{g}}_{c}’\left({\text{x}}_{i}\right)=\text{M}\left({\text{x}}_{i}\right)\;\cdot \;{\text{g}}_{c}\left({\text{x}}_{i}\right)+{\text{g}}_{c}\left({\text{x}}_{i}\right)$$ (1)

g (x_i_): the feature map of the Feature extractor

M (x_i_): the Attention map

g_c_’(x_i_): the feature map output by the Attention mechanism

{c | 1,…, C}: the number of implant brand types

#### 2.6.3. ABN training

ABN was learned end-to-end using the loss of attention branch and perception branch. The training loss function (L (x_i_)) of ABN was obtained by a simple addition of the training loss of the two branches as shown in the following equation.


$$\text{L}\left({\text{x}}_{i}\right)=L_{att}\left({\text{x}}_{i}\right)+L_{per}\left({\text{x}}_{i}\right)$$ (2)

L_att_ (x_i_): learning loss at attention branch

L_per_ (x_i_): learning loss at perception branch

The training loss of each branch is calculated using the softmax function and the cross-entropy. The ABN feature extractor is optimized by receiving the gradients of the attention branch and the perception branch.

### 2.7. Model training and procedure

Model training was generalized using k-fold cross-validation (k = 4) as a method of using all data other than test data in the training algorithm [22]. The test data consisted of 2034 images. Within each fold, the dataset was split into independent training sets and validation sets with a split ratio of 8:2. Each fold validation set is a fold that is completely independent of the other training folds and was used to assess training status during the training period. After completing one model training step, we performed similar validations four times using the test data with random seed.

All deep learning classification models were trained and evaluated on a 64-bit Ubuntu 18.04.5 LTS operating system (Canonical Ltd., London, UK) and an NVIDIA GeForce Tesla V100-SXM2 16 GB graphics processing unit (NVIDIA, Sta. Clara, CA, USA). All input image data were resized to 128 × 128 pixels. The optimizer, weight decay, and momentum were constant for all models. The optimizer used stochastic gradient descent, and the weight decay with momentum was 0.9. The learning rate was 0. 001. All the models were trained over a maximum of 100 epochs and a mini-batch size of 32. This process was repeated 30 times for both the simple CNN models and the ABN model using different random seeds.

### 2.8. Performance metrics

All the deep-learning models were evaluated in terms of different metrics: accuracy, precision, recall, specificity, and F1 score. These numerical performance metrics were calculated as in Eqs (3)–(7). In addition, we employed area under the curve (AUC) and receiver operating characteristics (ROC) metrics. Performance metrics were adopted to evaluate the model performance of the CNN.

In these equations, TP, FN, TN, FP represent true positive, false positive, true negative, and false negative respectively.


$$\text{Accuracy}=\frac{\text{TP}+\text{TN}}{\text{TP}+\text{FP}+\text{TN}+\text{FN}}$$ (3)

$$\text{Precision}=\frac{\text{TP}}{\text{TP}+\text{FP}}$$ (4)

$$\text{Recall}=\frac{\text{TP}}{\text{TP}+\text{FN}}$$ (5)

$$\text{Specificity}=\frac{\text{TN}}{\text{TN}+\text{FP}}$$ (6)

$$\text{F}1\;\text{score}=2\times \frac{\text{precision}\times \text{recall}}{\text{precision}+\text{recall}}$$ (7)

### 2.9. Visualization of computer-assisted diagnostic system

It is important to visualize the rationale for image prediction by CNN. Gradient-weighted class activation mapping (Grad-CAM) targets CNN-based image recognition models [23] It is a method to give a judgment basis to the model by weighting the gradient with respect to the predicted value. We used a heat map to emphasize the part that served as the basis for judgment according to the importance. Grad-CAM used the final convolution layer of the ResNet model, and the attention map visualization was reconstructed using the final layer of the attention branch.

### 2.10. Statistical analysis

A statistical evaluation of the classification performance between the simple CNN models and the ABN model was performed on the data. All data were analyzed 30 times repeatedly with a commercially available software, the JMP statistical software package version 14.2.0 for Macintosh (SAS Institute Inc., Cary, NC, USA) and *p-values* < 0.05 were considered statistically significant in all analyses. Non-parametric tests were performed based on the results of the Shapiro–Wilk test. The difference in classification performance between the simple CNN models and the ABN model was calculated for each performance metric by the Wilcoxon test. For multiple test comparisons in each model, Dunnett’s test was performed. Effect sizes were calculated as Hedges’ g (unbiased Cohen’s d) with the following equations.


$$Hedges\prime \;g=\frac{\left|M_{1}-M_{2}\right|}{s}$$ (8)

$$s=\sqrt{\frac{(n_{1}-1)s_{1}^{2}+\left(n_{2}-1\right)s_{2}^{2}}{n_{1}+n_{2}-2}}$$ (9)

M_1_ and M_2_ are the means each performance metrics for the simple CNN models and the ABN model, respectively; s_1_ and s_2_ are the standard deviations for the simple CNN models and the ABN model, respectively; and n_1_ and n_2_ are the numbers for the simple CNN models and the ABN model, respectively. The effect size determination was based on the following criteria proposed by Cohen and expanded by Sawilowsky [24]: huge effect was 2.0, very large effect was 1.0, large effect was 0.8, medium effect was 0.5, small effect was 0.2 and very small effect was 0.01.

## 3. Results

### 3.1. Comparison of the simple ResNet model and ABN model in classification performance

The results of comparing the simple ResNet models and the ABN models are presented in Table 4. In ResNet 18, the ABN model showed much higher classification performance compared to the simple CNN model. Interestingly, all performance metrics showed a statistically significant increase in ABN model, and the effect size was huge. On the other hand, both ResNet 50 and 152, simple CNN models had statistically significantly higher classification performance compared to the ABN model. With ResNet50, the effect size was equivalent to large in all performance metrics, whereas with ResNet 152, the effect size was large for all performance metrics except AUC, and the effect size for AUC was huge.

**Table 4:** Comparing each simple ResNet model and ABN model.

	Accuracy	Precision	Recall	F1 score	AUC
	SD	SD	SD	SD	SD
	95%CI	95%CI	95%CI	95%CI	95%CI
ResNet18	0.9486	0.9441	0.9333	0.9382	0.9979
	0.0026	0.0036	0.0039	0.0037	0.0003
	0.9460–0.9513	0.9404–0.9477	0.9293–0.9372	0.9345–0.9418	0.9976–0.9982
ResNet18 with ABN	0.9719	0.9686	0.9627	0.9652	0.9993
	0.0026	0.0036	0.0039	0.0037	0.0003
	0.9696–0.9741	0.9659–0.9714	0.9598–0.9656	0.9625–0.9678	0.9991–0.9994
P value	< 0.001	< 0.001	< 0.001	< 0.001	< 0.001
Effect size	9.300	7.488	8.402	8.330	6.361
ResNet50	0.9578	0.9546	0.9471	0.9498	0.9983
	0.0042	0.0047	0.0050	0.0048	0.0006
	0.9536–0.9619	0.9499–0.9593	0.9421–0.9521	0.9450–0.9546	0.9977–0.9989
ResNet50 with ABN	0.9511	0.9477	0.9382	0.9416	0.9975
	0.0028	0.0037	0.0049	0.0043	0.0002
	0.9483–0.9539	0.9440–0.9514	0.9333–0.9431	0.9374–0.9459	0.9973–0.9977
P value	< 0.001	< 0.001	< 0.001	< 0.001	< 0.001
Effect size	1.849	1.604	1.773	1.772	1.769
ResNet152	0.9624	0.9575	0.9509	0.9530	0.9985
	0.0039	0.0048	0.0052	0.0052	0.0009
	0.9584–0.9663	0.952–0.963	0.944–0.958	0.947–0.959	0.9984–0.9986
ResNet152 with ABN	0.9564	0.9514	0.9450	0.9470	0.9955
	0.0025	0.0032	0.0030	0.0028	0.0002
	0.9539–0.9588	0.9483–0.9546	0.9458–0.9561	0.9478–0.9582	0.9950–0.9957
P value	< 0.001	< 0.001	< 0.001	< 0.001	< 0.001
Effect size	1.803	1.458	1.399	1.403	3.725

SD: standard deviation, 95% CI: 95% confidence interval, AUC: Area under the ROC curve.

### 3.2. Comparison of multiple tests in each simple CNN model and ABN model

Following the comparison of the simple CNN and ABN models, we performed multiple comparisons of the performance metrics of the CNN models (ResNet18, 50, 152) with different layers in the simple CNN and ABN models and the results are shown in Table 5.

**Table 5:** Multiple comparisons of models with different numbers of layers for simple CNNs and CNNs with ABN; statistical results analyzed by Dunnett’s test.

Statistical results analysed by Dunnett test					
Performance metrics	Model B	Model A	A-B	p value	Effect size
only CNN					
Accuracy	ResNet152	ResNet50	-0.0091	< .0001	1.734
	ResNet18		0.0046	< .0001	3.324
Precision	ResNet152	ResNet50	-0.0105	< .0001	0.832
	ResNet18		0.0029	0.004	2.828
Recall	ResNet152	ResNet50	-0.0138	< .0001	0.941
	ResNet18		0.0039	0.001	3.051
F1 score	ResNet152	ResNet50	-0.0116	< .0001	0.876
	ResNet18		0.0032	0.002	2.881
AUC	ResNet152	ResNet50	-0.0004	< .0001	1.097
	ResNet18		0.0002	0.0007	1.584
CNN with ABN					
Accuracy	ResNet152	ResNet18	-0.0075	< .0001	4.737
	ResNet50		-0.0127	< .0001	6.091
Precision	ResNet152	ResNet18	-0.0086	< .0001	4.310
	ResNet50		-0.0123	< .0001	5.353
Recall	ResNet152	ResNet18	-0.0080	< .0001	4.174
	ResNet50		-0.0148	< .0001	5.939
F1 score	ResNet152	ResNet18	-0.0087	< .0001	4.310
	ResNet50		-0.0141	< .0001	5.994
AUC	ResNet152	ResNet18	-0.0034	< .0001	5.032
	ResNet50		-0.0014	0.0024	4.236

AUC: Area under the ROC curve.

For simple CNN models, Dunnett’s test was performed based on ResNet 50. ResNet50 achieved statistically significantly higher performance than ResNet18 in all performance metrics. Similarly, ResNet 152 performed statistically significantly better than ResNet 50. The results of the ABN model differed from those of the simple CNN models. In Dunnett’s test based on ResNet 18 with ABN, very interestingly, ResNet 18 with ABN showed statistically significantly higher performance than ResNet 50 and 152 with ABN.

### 3.3. Visualization of each model classification by Grad-CAM and attention heatmap

**Fig 2:**
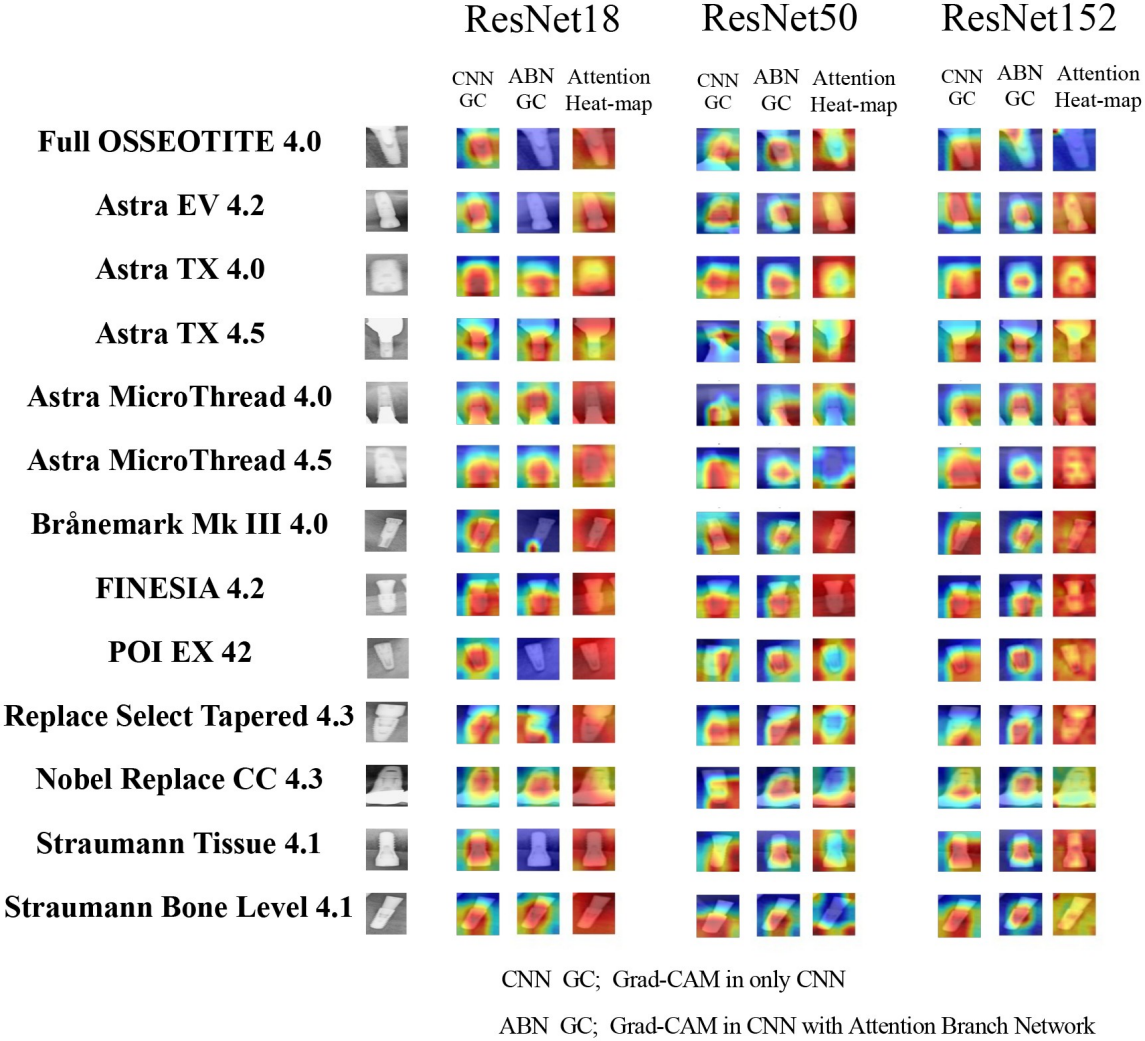
Visualization of the CNN’s region of interest using simple CNNs and ABNs with Grad-CAM and attention heatmaps.

Fig 2 shows the image that visualizes the region of interest of the deep learning model. The simple CNN models visualized the feature areas for implant classification from the final layer of the convolutional layer using Grad-CAM. The ABN model visualized the feature regions extracted from the feature extractor using Grad-CAM. Furthermore, the feature area obtained from the attention mechanism was visualized as an attention heat map. In feature extraction using the simple CNN models and Grad-CAM, there was no clear difference among the models. On the other hand, there was a very interesting discovery in the ABN model. In ResNet18, the features extracted from the feature extractor were not enough. However, the attention mechanism completely complemented the features. In contrast, the feature extractor enabled sufficient feature extraction with ResNet50 and 152. However, with ResNet50 and 152, it was confirmed that feature extraction became insufficient by going through the attention mechanism.

## 4. Discussion

This study compared dental implant classification performance using simple CNN models and an ABN model. ResNet18 with ABN was found to show the highest classification performance despite the small number of parameters.

There have been many studies on the classification of dental implants using CNNs [10–14, 16, 25] and these studies have achieved high classification accuracy. However, it is difficult to simply compare classification performance with previous studies because it was analyzed under different conditions such as the number of training and test images, the type of implant, and the model. In the past, three to twelve different types of implant classifications have been considered. In this study, 13 types of implant classification tasks were performed, which are more than those of the previous studies. In general, the accuracy decreases as the number of classifications increases. In our previous study [16], 12 classification tasks were 0.9787, 0.9800, 0.9851 accuracy for ResNet18, 50, and 152, respectively. In this study of 13 classifications, ResNet18, 50, and 152 were 0.9486, 0.9578, and 0.9624, respectively, and the classification performance deteriorated. However, performing many types of classification is a condition that more reflects clinical medicine, and the significance of our study will be great.

In the past, degradation occurred, and consequently, the accuracy became saturated and the performance was lower than that of a shallow network when the layer was deepened in deep learning. To address that issue, ResNet was developed with a shortcut added to the network [21]. Owing to this structure, the deep learning model could be more accurate as the layer was deeper. Even in the results of this study, ResNet152 achieved the best classification performance in a simple ResNet-only structure. In addition, statistical evaluation from the results of 30 repetitions showed significantly higher performance. In contrast, the results were very interesting in the ABN model. ResNet18 with ABN showed the highest performance. Surprisingly, the multiple comparison statistical evaluation among CNN models in each performance metric was also significantly better than ResNet 50 and 152 with ABN. The ABN model for dental implant classification revealed that it was not related to the depth and accuracy of the model’s layers.

In comparison between the simple CNN models and the ABN model, the ABN model had statistically higher performance metrics in ResNet18 and all effect sizes were huge. In contrast, in ResNet50 and 152, the ABN model had statistically lower performance metrics and all effect sizes were from large to huge. In the ABN model, the accuracy of the model was dramatically improved by adding an attention mechanism to ResNet18. In contrast, with ResNet 50 and 152, the addition of an attention mechanism reduced accuracy. One answer to this result was the visualization of the judgment area of the CNN. With ResNet 50 and 152, feature extraction with the feature extractor was sufficient to extract implants. The attention heatmap for ResNet 152 showed some deficiencies in the extraction of complete features. In ResNet50, the part opposite to the feature extraction in the feature extractor was judged as a feature in the attention heat map. This was thought to be due to the antagonism between the attention mechanism and the feature extraction. In ResNet18, some feature extractions were insufficient. However, in the attention heat map, the entire area was judged as the feature area. It has been reported that not only the implant fixtures but also the surroundings are the focus of judgment as the basis for judgment of implants by deep learning [12]. Therefore, with ResNet18, it is thought that higher classification accuracy could be achieved by focusing on the entire area. Few studies have reported using ABN for medical imaging. Ding et al. analyzed CNN models using ABN for skin lesion classification [26] and reported that the use of ABN significantly improved the classification accuracy of Melanoma and Seborrheic Keratosis. However, there are no reports on other medical images yet. Our study is the first to consider the use of ABN in dental implant classification.

In this study, effect size was also calculated as an evaluation method for model comparisons in deep learning. Effect size represents the effect of experimental manipulation and the strength of association between variables and is an index showing the degree of standardized effect that does not depend on the unit of data [27]. Effect size reporting, in contrast to its statistical significance, facilitates the interpretation of the importance of study results [28]. In this study, ResNet 18 with ABN showed significantly better performance at p-values statistically. Furthermore, when examining effect sizes, the ResNet18 model with ABN showed effect sizes that could be classified as very large effects. This suggests that a significant contribution of the model with ABN in implant identification. We are confident that our study is the first to show effect size in implant classification using deep learning with ABN and will play an important role in future studies.

Our research will provide an important perspective for implant diagnosis in future dental treatments. In the classification of dental implants, it is required to make a more accurate diagnosis with a model that is as light as possible. In this study, the ResNet18-based ABN model with the smallest number of parameters achieved the best accuracy. This result is very important. The fact that the addition of the attention mechanism did not significantly increase the number of parameters indicates that data processing does not require a high degree of computer processing power. In addition, the acquisition of performance metric variability from 30 independent tests proved a more accurate and generalized model. In future, our proposed implant classification model may become an effective model for clinical use.

In this study, ABN was used to perform an accurate classification of implant brands. Other implant-related details are also important for clinicians, dentists, and dental staff. The panoramic radiograph images should be helpful for locating the implant, classifying the brand, classifying the diameter of the implant, and conditioning the bone around the implant without cropping the image. Therefore, in the future, a deep-learning model for object detection should be implemented by using region-based convolutional neural networks, etc., to conduct accuracy verification research. The major dental implant systems vary in different parts of the world. Therefore, it is important to first create an accurate database for each implant. In order to enhance the global implant database, it is necessary to manage access to effective big data while adhering to medical ethics. After establishing the rules, deep learning using big data will help to build a powerful dental implant classification method and will contribute to dental care in the world.

This study has two limitations. The first is the type of CNN models we considered. There are so many well-known CNN models today that we need to validate a CNN model that is more compatible with the ABN. However, it requires a very large amount of calculation cost to obtain the effect size, and it is hoped that you will refer to our examination this time. The second is the state of the used image data. The image used in this study is removed from the implant as a pretreatment. With ABN, we achieved a very high classification performance when considering using this image. However, in the future, it is desirable to be able to identify the site where the implant is implanted from the panoramic radiograph and further classify the type of implant. Further research is required from this point of view as well.

## 5. Conclusions

In this study, we proposed an optimal deep learning model for comparing the classification performance of dental implants using a simple CNN model and an ABN model. In the dental implant classification, we found that ResNet 18 showed very high compatibility in the ABN model and showed the best classification performance despite the small number of parameters. These results may play an important role in the rapid and accurate classification of dental implants in clinical dental settings.

## Supporting information

S1 Fig Mean ROC curves of simple CNNs and CNNs with ABN models for 13 types of dental implant classification. (DOCX)

S2 Fig Visualization of each model classification by Grad-CAM and attention heatmap in ResNet18. (DOCX)

S3 Fig Visualization of each model classification by Grad-CAM and attention heatmap in ResNet50. (DOCX)

S4 Fig Visualization of each model classification by Grad-CAM and attention heatmap in ResNet152. (DOCX)
