# Supplementary material for: Is attention branch network effective in classifying dental implants from panoramic radiograph images by deep learning?
Source: PLoS One. 2022 Jul 27;17(7):e0269016. doi: 10.1371/journal.pone.0269016 (PMC9328496; doi:10.1371/journal.pone.0269016)

Is attention branch network effective in classifying dental implants from panoramic radiograph images by deep learning?

Shintaro Sukegawa ^1,2,^ *, Kazumasa Yoshii ^3^, Takeshi Hara ^4,5^, Futa Tanaka ^4^, Katsusuke Yamashita ^6^, Tutaro Kagaya ^3^, Keisuke Nakano ^2^, Kiyofumi Takabatake ^2^, Hotaka Kawai ^2^, Hitoshi Nagatsuka ^2^, Yoshihiko Furuki ^1^

^1^ Department of Oral and Maxillofacial Surgery, Kagawa Prefectural Central Hospital, 1-2-1, Asahi-machi, Takamatsu, Kagawa, Japan

^2^ Department of Oral Pathology and Medicine, Graduate School of Medicine, Dentistry and Pharmaceutical Sciences, Okayama University, Okayama, Okayama, Japan

^3^ Department of Intelligence Science and Engineering, Graduate School of Natural Science and Technology, Gifu University, Gifu, Gifu, Japan

^4^ Department of Electrical, Electronic and Computer Engineering, Faculty of Engineering, Gifu University, Gifu, Gifu, Japan

^5^ Department of Intelligence Science and Engineering, Graduate School of Natural Science and Technology, Gifu University, Gifu, Japan

^6^ Polytechnic Center Kagawa, Takamatsu, Kagawa, Japan

***Corresponding Author**

Email address: [gouwan19@gmail.com](mailto:gouwan19@gmail.com) (SS)

**S2 Fig. Visualization of each model classification by Grad-CAM and Attention heatmap in ResNet18**

**Visualization using Grad-Cam of ResNet18 and Grad-CAM and Attention heatmap of ResNet18 with ABN**


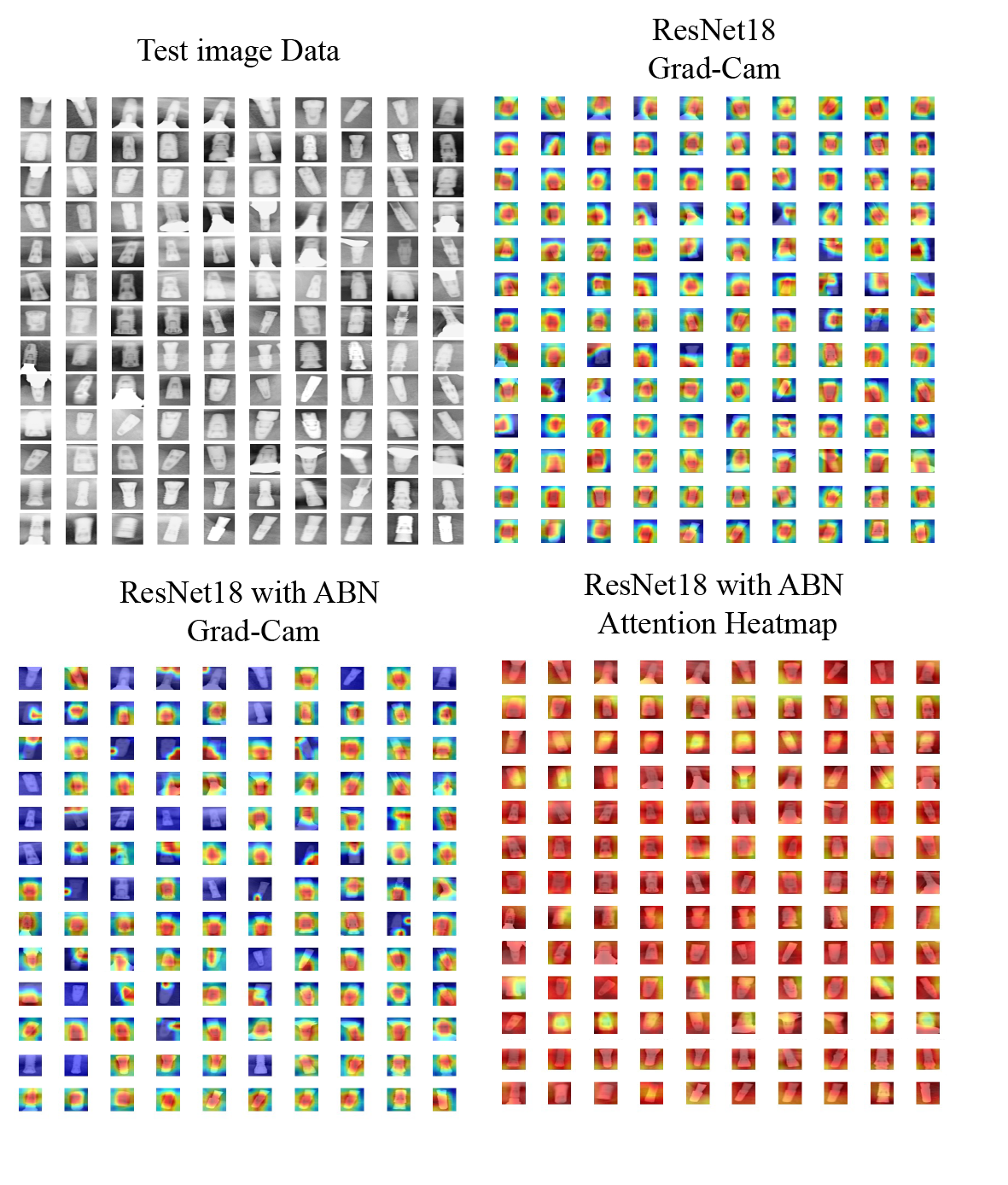

Supplement: S2 Fig — (DOCX) [file pone.0269016.s002.docx]
